# Modeling Cell-Cell Interactions from Spatial Molecular Data with Spatial Variance Component Analysis

**DOI:** 10.1016/j.celrep.2019.08.077

**Published:** 2019-10-01

**Authors:** Damien Arnol, Denis Schapiro, Bernd Bodenmiller, Julio Saez-Rodriguez, Oliver Stegle

**Affiliations:** 1 European Molecular Biology Laboratory, European Bioinformatics Institute, Wellcome Genome Campus, Hinxton, Cambridge CB10 1SD, UK; 2 Joint Research Center for Computational Biomedicine, RWTH Aachen University, Faculty of Medicine, Pauwelsstrasse 19, 52074 Aachen, Germany; 3 European Molecular Biology Laboratory, Genome Biology Unit, Heidelberg, Germany; 4 Institute of Molecular Life Sciences, University of Zurich, Zurich, Switzerland; 5 Life Science Zurich Graduate School, ETH Zurich and University of Zurich, Zurich, Switzerland; 6 Institute for Computational Biomedicine, Heidelberg University, Faculty of Medicine, Bioquant, 69120 Heidelberg; 7 Division of Computational Genomics and Systems Genetics, German Cancer Research Center, 69120 Heidelberg, Germany

**Keywords:** Gaussian process, random effect model, multiplexed imaging

## Abstract

Technological advances enable assaying multiplexed spatially resolved RNA and protein expression profiling of individual cells, thereby capturing molecular variations in physiological contexts. While these methods are increasingly accessible, computational approaches for studying the interplay of the spatial structure of tissues and cell-cell heterogeneity are only beginning to emerge. Here, we present spatial variance component analysis (SVCA), a computational framework for the analysis of spatial molecular data. SVCA enables quantifying different dimensions of spatial variation and in particular quantifies the effect of cell-cell interactions on gene expression. In a breast cancer Imaging Mass Cytometry dataset, our model yields interpretable spatial variance signatures, which reveal cell-cell interactions as a major driver of protein expression heterogeneity. Applied to high-dimensional imaging-derived RNA data, SVCA identifies plausible gene families that are linked to cell-cell interactions. SVCA is available as a free software tool that can be widely applied to spatial data from different technologies.

## Highlights

• Statistical method to assess cell-cell interactions in spatial expression data
• Generally applicable to diverse data types and biological systems
• Illustrated on IMC data in human cancer and seqFISH data in mouse hippocampus
• Open source software available on github

## Graphical Abstract

**Figure:**
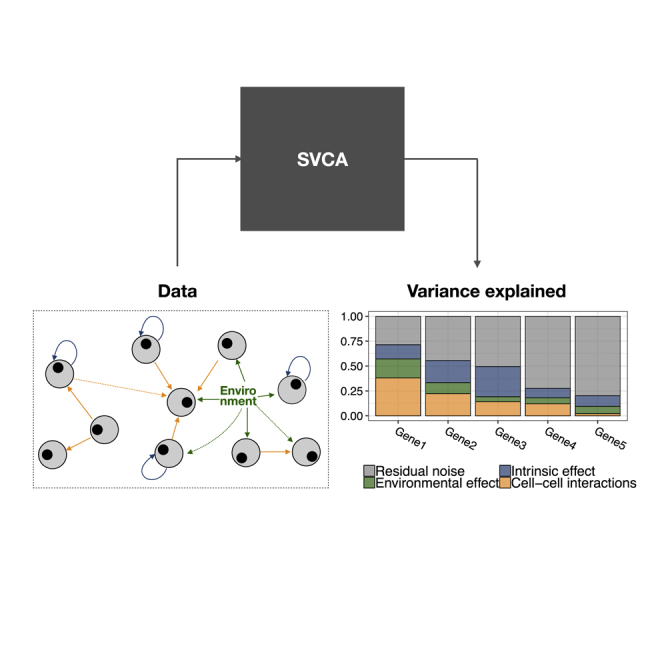

## Teaser

Arnol et al. present a statistical method for analyzing single-cell expression data in a spatial context. The method identifies the sources of gene expression variability by decomposing it into different components, each attributable to a different source. These sources include aspects of spatial variation, in particular cell-cell interactions.

## Introduction

Experimental advances enable assaying RNA and protein abundances of single cells in spatial contexts, thereby allowing the study of single-cell variations in tissue. Already, these technologies have delivered insights into the spatial structure of cell types in tissue and its effect on gene expression programs (Bodenmiller, 2016, Battich et al., 2013). These new dimensions of gene expression variation also have the potential to deliver biomarkers in health and disease (Bodenmiller, 2016).

Currently, there exist alternative technologies for profiling spatially resolved expression profiles. Imaging Mass Cytometry (IMC) (Giesen et al., 2014, Chang et al., 2017) and Multiplexed Ion Beam Imaging (MIBI) (Angelo et al., 2014) rely on protein labeling with antibodies coupled to metal isotopes of specific masses followed by high-resolution tissue ablation and ionization. IMC currently allows for assaying of up to 37 targeted proteins with subcellular resolution. Alternative methods such as multiplex immunofluorescence (MxIF) and cyclic immunofluorescence (CycIF) use immunofluorescence for protein quantification of dozens of markers in single cells (Gerdes et al., 2013, Lin et al., 2015). There are also rapidly evolving technologies based on fluorescence assays to measure single-cell RNA levels in spatial context (Strell et al., 2018). Among these, multiplexed error robust-fluorescence *in situ* hybridization (Mer-FISH) and sequential FISH (seqFISH) use a combinatorial approach of fluorescence-labeled small RNA probes to identify and localize single RNA molecules (Shah et al., 2017, Chen et al., 2015, Gerdes et al., 2013, Lin et al., 2015), which has dramatically increased the number of readouts (currently between 130 and 250). Even higher-dimensional expression profiles can be obtained from spatial expression profiling techniques such as spatial transcriptomics (Ståhl et al., 2016). However, they currently do not offer single-cell resolution and are therefore not sufficient for studying cell-to-cell variations.

The availability of spatially resolved expression profiles from a population of cells provides new opportunities to disentangle the sources of gene expression variation in a fine-grained manner. Spatial methods can be utilized to distinguish intrinsic sources of variation, such as the cell-cycle stages (Buettner et al., 2015, Scialdone et al., 2015), from sources of variation that relate to the spatial structure of the tissue, such as microenvironmental effects linked to the cell position (Fukumura, 2005), access to glucose or other metabolites (Meugnier et al., 2007, Lyssiotis and Kimmelman, 2017), or cell-cell interactions. To perform their function, proximal cells need to interact via direct molecular signals (Sieck, 2014), adhesion proteins (Franke, 2009), or other types of physical contacts (Varol et al., 2015). In addition, certain cell types such as immune cells may migrate to specific locations in a tissue to perform their function in tandem with local cells (Moreau et al., 2018). In the following we refer to cell-cell interactions as a general term regardless of the underlying mechanism, while more specific biological interpretations are discussed in the context of the specific biological use cases we present.

While intrinsic sources of variation have been extensively studied, cell-cell interactions are arguably less well explored, despite their importance for understanding tissue-level functions. Experimentally, the required spatial omics profiles can already be generated at high throughput, and hence there is an opportunity for computational methods that allow for identifying and quantifying the impact of cell-cell interactions.

Existing analysis approaches for spatial omics data can be broadly classified into two groups. On the one hand, there exist statistical tests to explore the relevance of the spatial position of cells for the expression profiles of individual genes (Svensson et al., 2018). Genes with distinct spatial expression patterns have also been used as markers to map cells from dissociated single-cell RNA sequencing (RNA-seq) to reconstructed spatial coordinates (Achim et al., 2015, Satija et al., 2015). However, these approaches do not consider cell-cell interactions.

On the other hand, there exist methods to test for qualitative patterns of cell-type organization. For example, recent methods designed for IMC datasets (Schapiro et al., 2017, Schulz et al., 2018) identify discrete cell types that co-occur in cellular neighborhoods more or less frequently than expected by chance. While these enrichment tests yield qualitative insights into interactions between cell types, these methods do not quantify the effect of cell-cell interactions on gene expression programs. Alternatively, there exist regression-based models to assess interactions on gene expression profiles of genes based on predefined features that capture specific aspects of the cell neighborhood (Goltsev et al., 2018, Battich et al., 2015). These models are conceptually closely related to our approach; however, they rely on the careful choice of relevant features and tend to require *ad hoc* discretization steps to define cell neighborhoods (see STAR Methods).

Here, we present spatial variance component analysis (SVCA), a computational framework based on Gaussian processes (Rasmussen and Williams, 2006), to model spatial sources of variation of individual genes. SVCA allows for decomposing gene expression variation into intrinsic effects, environmental effects, and, most importantly, an explicit cell-cell interaction component. In contrast to previous methods, our model directly uses the spatial coordinates and the gene expression profile of each cell as input, thereby avoiding the need to define discrete cell types and other microenvironmental variables.

We validate our model using simulated data, demonstrating the accuracy of the model and its robustness to technical sources of variation including mis-segmentation. We then apply SVCA to two datasets from different technologies and biological domains: IMC proteomics profiles data from human breast cancer tissue (Schapiro et al., 2017) and spatial single-cell RNA profiles from the mouse hippocampus generated using seqFISH (Shah et al., 2017). Across these domains, we find that the cell-cell interaction component in our model explains a major share of expression variability, thus facilitating the identification of biologically relevant genes and pathways that participate in cell-cell interactions.

## Results

### SVCA: A Statistical Framework for Decomposing Spatial and Non-spatial Sources of Variation

**Figure 1:**
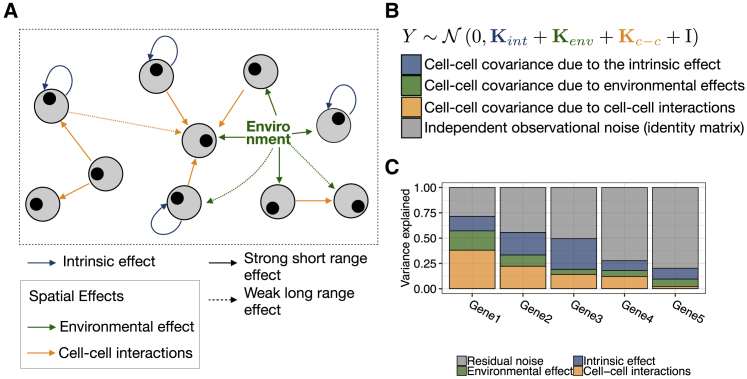
Spatial Variance Component Analysis (SVCA): A Framework for Decomposing Spatial and Non-spatial Sources of Variation (A) SVCA decomposes the variability of individual genes into (1) cell intrinsic effects (due to differences in intrinsic cell type or state, blue); (2) general environmental effects that capture expression differences due to non-specific local factors (green); and (3) a cell-cell interaction effects that capture differences in expression level attributable to different cellular composition of a cell’s neighborhood (yellow). (B) SVCA builds on the random effect framework to model additive contributions of these components. See Figure S1 and STAR Methods for details on the definition of the corresponding covariance terms. (C) SVCA output: gene-level breakdown of the proportion of variance attributable to different components.

SVCA builds upon the random effect framework to model gene expression variation of individual genes as a function of additive components of intrinsic cell state effects, $U_{int}$; an environmental effect linked to the cell position, $U_{env}$; and an effect due to cell-cell interactions, $U_{c-c}$: $Y=U_{int}+U_{env}+U_{c-c}+\epsilon .$ Here, Y denotes the vector of the expression levels of a gene of interest across all cells and $\epsilon ∼N\left(0,\sigma_{\epsilon }^{2}I\right)$ denotes Gaussian measurement noise. These random effects are assumed to follow multivariate normal distributions, defined by covariance matrices that are functions of the cell spatial positions and expression profiles: $U_{int\;}∼N\left(0,K_{int}\right)$, where $K_{int}$ is a covariance matrix that quantifies the pairwise similarity of cells in terms of their intrinsic state; $U_{env}∼N\left(0,K_{env}\right),$ where $K_{env}$ quantifies the similarity between the environmental context of cells based on their spatial proximity; and $U_{c-c}∼N\left(0,K_{c-c}\right)$, where $K_{c-c}$ measures the similarity between the cellular neighborhoods of cells, thereby accounting for cell-cell interactions. Equivalently, this model can be expressed as the joint normal distribution with additive covariance terms $Y=N\left(0,K_{int}+K_{env}+K_{c-c}+\sigma_{\epsilon }^{2}I_{n}\right)$ (Figure 1B).

The intrinsic cell-state covariance$K_{int}$is estimated based on the expression profiles of all genes except the focal gene: ${\left(K_{int}\right)}_{i,j}=\sigma_{int}^{2}X_{i}\cdot X_{j}^{T}.$ Here, $X_{i}$ and $X_{j}\;$denote the vectors of expression levels of all genes but the target gene in cell i and j, and $\sigma_{int}^{2}$ is a scaling parameter that is proportional to the variance explained by this covariance. The covariance for the environmental context $K_{env}$ is calculated based on the pairwise distance of all genes, $K_{env\;}=\sigma_{E}^{2}exp\left(-d_{i,j}^{2}/2l^{2}\right)$, where $d_{i,j}$ denotes the physical distance between cells i and j. This component captures differences in the (local) environment or technical drift in the measurement process. The cell-cell interaction covariance term $K_{c-c}$ quantifies the similarity of the cellular composition in the neighborhood of cells. Borrowing concepts from social genetic effect studies (Baud et al., 2017), we define this covariance by aggregating, for each cell, the molecular composition of all other cells weighted by their distance, $K_{c-c}=\sigma_{c-c}^{2}\;Z\cdot XX^{T}\cdot Z^{T}$. Here, Z is a matrix that defines the continuous neighborhood of each cell, weighted by an exponential decay with cell distances $Z_{i,j}=exp\left(-d_{i,j}^{2}/2l^{2}\right)$. Finally, the noise term captures the unexplained residual gene expression variation. Figure 1A provides a schematic overview of these different variance components in SVCA and Figure S1 presents further details on the definition of the covariance terms used by the model (see also the STAR Methods).

The SVCA model is fitted for every target gene using maximum likelihood, to determine the scaling parameters $\sigma_{I}^{2},\sigma_{E}^{2},\sigma_{c-c}^{2}$, and $\sigma_{\epsilon }^{2}$, as well as the length-scale parameter l. See Rasmussen and Williams (2006) for an overview of the parameter interface in this class of multivariate normal models. The fitted model can also be used to estimate the fraction of variance explained by each term after appropriate rescaling, using Gower factors (Searle, 1982, Kostem and Eskin, 2013; STAR Methods). This results in a breakdown for each gene of the fraction of variance explainable by spatial and non-spatial variance components, yielding a compact representation of major drivers of gene expression variation (Figure 1C). In the following we denote this representation as a spatial variance signature. Additionally, SVCA can be used to assess the statistical significance of individual variance terms, using model comparisons between the full SVCA model and reduced models in which individual covariance terms are omitted (STAR Methods). Finally, SVCA can also be used to predict expression profiles of held-out cells (STAR Methods).

Notably, SVCA does not require discrete cell-type assignments, but instead is based on continuous measures of cell-cell similarity that are directly estimated from cell expression profiles (Figure S1). The model also circumvents the need to define local cell neighborhoods, but instead weights interactions between pairs of cells as a function of their distance (Figure S1). Additionally, SVCA includes a non-linear environmental component which captures non-specific spatial effects. As we will observe later, this component captures unspecific variation that is linked to the location of a cell, including confounding factors such as technical drifts.

Initially, we used simulated data from the SVCA generative model to validate the model. We simulated expression profiles with no interaction effects to assess the calibration of the statistical test for cell-cell interactions, finding that the model yields conservative estimates (Figure S2A). We also compared the estimated variance components for cell-cell interactions with the simulated variance components when simulating increasing fractions of the variance explained by interaction effects, observing that the model yields accurate variance estimates (Figure S2B). We then assessed the power for detecting true cell-cell interactions, simulating increasing fractions of cell-cell interactions (Figure S2C), as well as the number of cells contained in the dataset (Figure S2D). To investigate the empirical identifiability of cell-cell interactions versus environmental effects, we also compared the estimates of the full model to a reduced model, without the cell-cell interaction component (Figure S2E). These results show that the environmental effect can falsely explain spatial variation if not accounted for by the cell-cell interaction term. This indicates that this component has the capacity to capture confounding effects by other spatial sources of variation, as we observed in the first round of simulations. Overall, we demonstrate that SVCA can be used to estimate and test for spatial drivers of single-cell variability, in particular cell-cell interactions.

### SVCA Yields More Accurate Cell Interaction Estimates than Alternative Models

**Figure 2:**
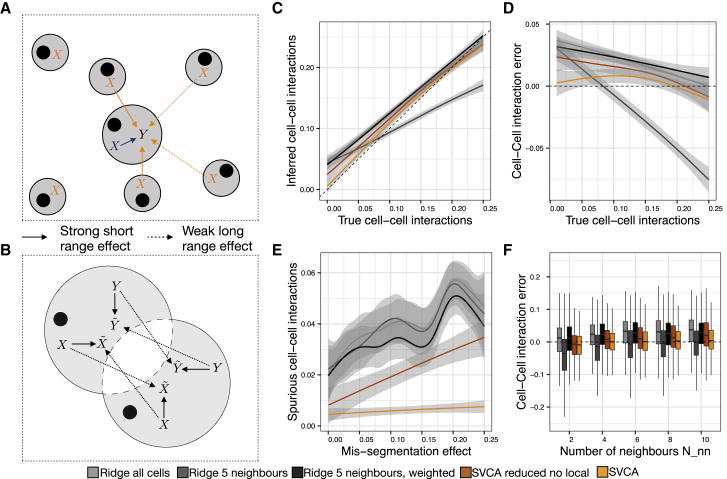
SVCA Is More Conservative and Robust than Alternative Linear Models (A) Simulation approach: the expression profile of a simulated target gene *Y* is generated as a linear combination of the empirically observed cell expression profile of all genes (*X*) and a linear combination of the $N_{nn}$ first neighbors expression profiles (*X*) (here, $N_{nn}$ = 4). The effect of the first neighbors is weighted by the function of their distance to the focal cell. (B) Simulation of cell mis-segmentation effects. Pairs of cells are randomly selected as mis-segmented with probability inversely proportional to the square of their distance (STAR Methods). (C) Inferred cell-cell interactions versus simulated true values for $N_{nn\;}=4$ and $\varepsilon_{mis\;}=0.2$. (D) Error in the inferred cell-cell interactions as a function of the simulated interaction component. (E) Spurious cell-cell interactions as a function of the simulated mis-segmentation effect (as in B). (F) Distribution of the cell-cell interaction error across as a function of the number of neighbors ($N_{nn}$).

Next, we considered a more complex simulation using empirical parameters derived from 11 real datasets, to compare SVCA to alternative models. Briefly, we stimulated gene expression profiles based on a linear model that accounts for intrinsic effects and cell-cell interactions of variable size, as well as confounding effects due to cell mis-segmentation (STAR Methods). Cell intrinsic effects were simulated as a linear combination of the empirical expression profile of all other genes: $X\cdot \beta_{I}$, where $\beta_{I}$ is a fixed effect size, and X is the matrix of expression profiles for all cells. Cell-cell interactions are simulated using a linear combination of the nearest-neighbor expression profiles, weighted by a function of the distance $Z\cdot X\cdot \beta_{c-c}$, where $\beta_{c-c}$ controls the size of the cell-cell interactions. $Z_{i,j}=1/d_{i,j}^{2}$ for the $N_{nn}$ nearest neighbors of all focal cells, and $Z_{i,j}=0$ otherwise ($d_{i,j}$ is the distance between cells i and j) (Figure 2A; STAR Methods). To simulate errors due to mis-segmentation, the generated expression profiles were perturbed by assigning a share of the expression profiles of mis-segmented neighboring cells, which results in perturbed expression profiles Xand Y. We varied the number of cell neighbors $N_{nn}$, the magnitude of the cell-cell interactions, and the extent of mis-segmentation effects (Figure 2B; STAR Methods).

We compared SVCA to four baseline methods: (1) a reduced random effect model, with the same covariance terms as SVCA but omitting the environmental term; (2) linear regression using the average of the expression profiles of the five nearest neighbors as input features; (3) linear regression accounting for cell-cell interactions between all pairs of cells weighted by the distance between cells; and (4) a combination of the last two methods, considering a fixed cellular neighborhood and weighting cell-cell interactions as a function of cell distance (STAR Methods). SVCA yielded the most accurate estimates of the cell-cell interaction component (Figures 2C and 2D) and the model was more robust to spurious effects due to mis-segmentation (spurious variance component below 1% for SVCA compared to up to 6% average for linear models; Figure 2E). The largest relative gains in accuracy were observed for small cell-cell interaction effects (Figure 2C). The lower accuracy of the reduced SVCA model with no environmental term indicates that this term plays an important role and in particular absorbs possible spurious effects from segmentation errors (spurious cell-cell interaction variance component of up to 3% versus < 1% and higher variance; Figure 2E). Additionally, SVCA was in general less biased across the full range of simulation settings than alternative methods (Figure 2F).

### Application of SVCA to a Breast Cancer Proteomics Dataset Identifies Cell-Cell Interactions as a Major Driver of Expression Variation

**Figure 3:**
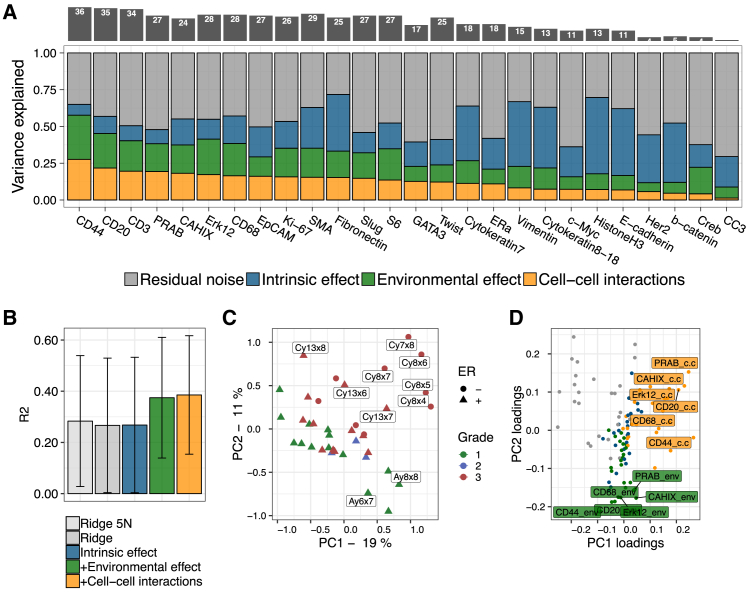
Application of SVCA to 46 Breast Cancer Samples Profiled Using IMC (A) Bottom panel: SVCA signatures for 26 proteins. Shown are averages of the fraction of variance explained by intrinsic effects, environmental effects, and cell-cell interactions, across 46 images. Proteins are ordered by the magnitude of the cell-cell interaction component. Top panel: number of images with a cell-cell interaction component greater than 10% variance. (B) Accuracy of SVCA and alternative models for predicting gene expression out of sample (r^2^ assessed using 5-fold cross validation). Shown are average coefficients of determination (r^2^) between predicted and observed gene expression profiles, averaged across proteins and images. Error bars correspond to ±1 SD across images and proteins. (C) First two principal components for 38 images with clinical annotations, calculated based on the spatial variance signature (variance break down as in A for each protein), with individual images colored by the clinical tumor grade. (D) Loadings of the principal components as in (C), displaying the relevance of individual proteins and types of variance components.

Next, we applied SVCA to an IMC dataset from human breast cancer, where 26 protein expression levels were quantified at the single-cell level in 46 breast cancer biopsies (Schapiro et al., 2017). SVCA revealed substantial differences of the overall importance of cell-cell interaction components across proteins, explaining up to 25% of the total expression variance on average (Figure 3A). Immune cell markers in particular were identified among the set of proteins with the largest cell-cell interaction effects: CD44, CD20, CD3, and CD68, for which cell-cell interaction explained more than 10% of the variance in 36, 35, 34, and 28 out of the 46 images, respectively (Figure 3A). We hypothesize that this effect could reflect the recruitment of immune cells by specific cellular environments (Moreau et al., 2018, Chlon and Markowetz, 2017). CAHIX, a marker of hypoxia, was also found among the top markers linked to cell-cell interaction effects. We confirmed the consistency of the variance estimates from SVCA using cross-validation, where SVCA yielded more accurate out-of-sample gene expression imputations than alternative regression models, as well as simplified models that ignore cell-cell interactions (Figures 3B and S3; STAR Methods). As an additional sanity check, we also compared the variance estimates to results obtained after permuting the cell positions, which as expected resulted in near-zero cell-cell interaction components (Figure S3).

We also observed substantial variation of the estimated spatial variance signatures between images (Figure S3), motivating investigation of the relationship between spatial variance components and clinical covariates, including tumor grade. A projection of the full SVCA output (spatial variance signature; Figure 3A) using principal-component analysis (PCA) identified the substructure between images that was significantly aligned with tumor grade (Figure 3C; p = 3.8 × 10^–3^; STAR Methods). Inspection of the PCA loadings (Figure 3D) identified the cell-cell interaction component and the environmental component for a subset of proteins (including CD20 and CD44) as the most informative SVCA features for PC1, which correlates with tumor grade. We also noticed that the images with the strongest separation in the PCA representation (image names highlighted in Figure 3C) have previously been highlighted in the primary analysis of this dataset, where these images were identified as exhibiting a different tissue organization compared to other images (Schapiro et al., 2017). This study also considered a permutation-based approach to identifying cell types that are enriched or depleted for co-occurence, followed by hierarchical clustering in order to detect images with similar cellular neighborhood structures. As a result of this procedure, the highlighted images were separated in a grade-1-enriched cluster containing the images Ay6x7 and Ay8x8 and a grade-3-enriched cluster containing the images Cy7x8, Cy8x4, Cy8x5, Cy8x6, Cy8x7, Cy13x6, Cy13x7, and Cy13x8 (Figure 3C) (Schapiro et al., 2017). This indicates that SVCA signatures capture variations that are identified using classical neighborhood statistics. Importantly, however, SVCA does not rely on cell-type classification and does not require a predefined definition of cell neighborhoods.

Tumor progression is characterized by disorganization and irregular cellular architecture, which is associated with larger cells, increased proliferation, and thus higher cell density in comparison to healthy breast tissue (Elston and Ellis, 1991). We investigated how SVCA signatures are affected by these environmental features and discovered a significant correlation (linear regression; p = 3.0 × 10^–3^) between the average number of neighbors per cell and the average cell-cell interaction components across proteins (using cellProfiler to estimate the number of cells). This relationship may in part explain the separation by tumor grade. In general, it is not surprising that the magnitude of cell-cell interactions is higher in tissue with increased cell density compared to adipose tissue with sparse cell coverage.

### Application of SVCA to an Hippocampus RNA Dataset Identifies Relevant Gene Families Involved in Cell-Cell Interactions

**Figure 4:**
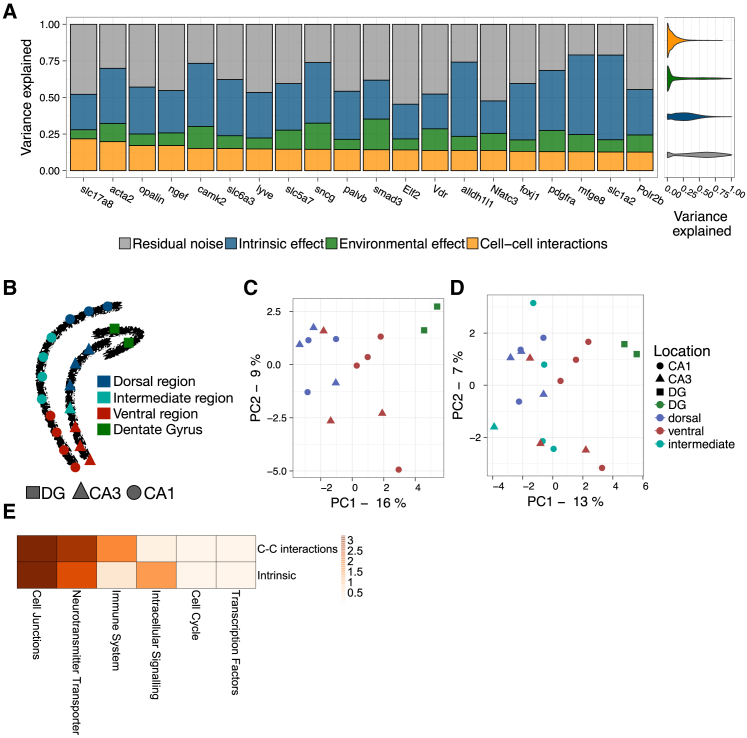
Application of SVCA to 21 Images Profiled Using seqFISH (A) Left: SVCA signatures for the 20 genes with the largest cell-cell interaction component. Shown are averages of the fraction of variance explained by intrinsic effects, environmental effects, and cell-cell interactions, across 21 images. Genes are ordered by the magnitude of the cell-cell interaction component. Right: variance estimate distribution across images and genes for all 249 genes contained in this dataset (violin plots). (B) Spatial organization of the mouse hippocampus with dots corresponding to individual images. Colors and shapes denote regions using the classification as in Shah et al. (2017). (C) First two principal components of the spatial variance signatures for individual images from the DG, the dorsal region, and the ventral region. Color and shape represent the location of the biopsy in the hippocampus. (D) First two principal components of the spatial variance signatures for all 21 images. (E) Enrichment of gene categories for cell-cell interactions (top) and intrinsic effect (bottom) (negative log Benjamini-Hochberg adjusted p values).

SVCA can be used for the analysis of data from a broad range of spatially resolved technologies, including optical-imaging-based assays. To explore this, we considered a mouse hippocampus dataset profiled using seqFISH (Shah et al., 2017), in which 249 RNA expression levels were assayed in 21 distinct brain regions of a single animal. Spatial variance signatures for the 20 genes with the largest cell-cell interaction component are shown in Figure 4A. Analogous to the IMC dataset, SVCA signatures were robust and models that account for cell-cell interactions yielded more accurate gene expression predictions (Figure S4).

Similarly to results obtained from the IMC datasets, we observed differences in the spatial variance signatures across images, which were sampled from functionally distinct regions of the hippocampus (Shah et al., 2017). Principal components of the spatial variance signature for the dorsal region clustered together, irrespective of their CA1/CA3 location (Figure 4B). Similarly, images from the dentate gyrus (DG) also clustered together, and there was some proximity between signatures from the ventral region, although with more variation between them (Figures 4C and 4D). This is consistent with the observation by Shah et al. (2017) that the ventral and dorsal regions of the CA1 and CA3 mirror each other with respect to their cellular compositions and ventral regions are more heterogeneous in their cellular composition. Spatial variance signatures for intermediate regions, however, did not show much resemblance (Figure 4D).

Leveraging the higher dimensionality of these data, we sought to identify gene families that participate in cell-cell interactions. First, we manually classified genes into non-overlapping categories based on prior annotations (Table S1), considering categories with more than five genes, including genes involved in the cell cycle, cell junctions, the immune system, neurotransmitter transporters, and transcription factors for further analysis. The neurotransmitter transporter category consisted of six glutamate transporters of the solute carrier family (slc genes; Masson et al., 1999, Iversen, 2006). The immune system category consisted of six genes with multiple functions, consistently associated with immune response, such as MFGE8, which is involved in phagocytosis, or the interferon regulatory factor IRF2. The eight-cell-junction genes included ACTA2 (Actin), Opalin (Yoshikawa et al., 2008), and MOG. The largest group was made up of annotated transcription factors, consisting of 166 genes.

We tested which of these categories are enriched for large cell-cell interaction components (STAR Methods), finding that cell junction genes and neurotransmitter transporters were the most enriched groups (Q = 6 × 10^–4^ and Q = 1 × 10^–3^, Benjamini Hochberg adjusted across gene sets) (Figure 4E). Individual cell junction genes, such as GJA1 (connexin), are involved in gap junction intercellular communication (Cheng et al., 2015), while, for example, the actin skeleton has a known role in the adaptation of tissue structure and geometry to external stimulus (Carpenter, 2000, Brakebusch and Fässler, 2003). This may explain why the single-cell expression levels of cell junction genes appeared to be regulated by cell-cell interactions. The enrichment of glutamate transporters is also consistent with their involvement in the transport and (re)uptake of the neurotransmitter at the neuronal synapses, a critical cell-cell interaction in the brain (Masson et al., 1999, Iversen, 2006, Angulo et al., 2004, Mason, 2017). In addition, Slc5a7 (CHT) was also found to be preferentially expressed in specific interneurons with a link to the spatial organization of the tissue (Yi et al., 2015). To a smaller extent, genes related to the immune system were enriched for cell-cell interactions (Q = 2 × 10^–2^). Among the top cell-cell-interaction-related genes were CTSS (Cathepsin) and MFGE8 (Lactadherin), which play a role in phagocytosis in the brain, a form of cell-cell interaction (Fricker et al., 2012, Neher et al., 2013, Vitner et al., 2010). Notably, however, cell junction genes and neurotransmitter transporters were also enriched among genes with high intrinsic effect, suggesting that the expression level of these genes also relates to intracellular processes.

Five out of the ten genes with the highest cell-cell interaction variance components did not fall into any of the considered gene set categories. NGEF (Ephexin) is an exchange factor that plays a role in axon guidance (Shamah et al., 2001, O’Donnell et al., 2009), CAMK2 is a kinase shown to play a role in long-term potentiation and neurotransmitter release (Wang, 2008, Lisman et al., 2012), LYVE is a membrane receptor (Banerji et al., 1999), and SNCG (synuclein gamma) is involved in axonal architecture (Surguchov et al., 2001, Vargas et al., 2017). Taken together, this shows that genes with large cell-cell interaction components, as identified using SVCA, have known implications in cell-cell communication between neurons, or have known annotations for regulating the spatial architecture of the tissue.

## Discussion

We have presented SVCA, a regression-based framework for the analysis of spatially resolved molecular expression data. Our model computes a spatial variance signature for individual mRNA or protein levels, decomposing their sources of variation into spatial and non-spatial components. Most prominently, SVCA provides a quantitative assessment of the effect of cell-cell interactions on the expression profile of individual molecules. SVCA tackles the problem of cellular classification and neighborhood definition using a continuous representation of space and cellular identity (Wagner et al., 2016).

We have applied SVCA to multiple datasets generated using alternative technologies, probing either RNA transcripts or proteins, demonstrating the broad applicability of the approach. Across these applications, we observed that cell-cell interactions can substantially contribute to gene expression variation, which is consistent with previous reports (Battich et al., 2015, Goltsev et al., 2018, Kamińska et al., 2015, Ayuob and Ali, 2012) and supports the concept that studying single-cell expression in the native context is important for understanding the sources of these variations.

We noticed variation in the SVCA signatures across images and investigated the possible causes of this variability. We provided evidence that differences in SVCA signatures could result from differences in the spatial structure of tissue, as well as different clinical and biological contexts. For the IMC data, we also noticed that this variability reflected previous findings about different tissue organizations between samples.

We used gene annotation to interpret the spatial variance signatures of individual genes and pathways. This identified genes with known involvements in cellular interactions, even specific to the brain, such as SLCs, to be predominantly enriched in the corresponding terms of our models. In addition to confirming the biological relevance of SVCA signatures, these results suggest that spatial variance signatures can be utilized to study the involvement of individual genes in tissue-level functions. Further interpretation of these signatures, in particular of the cell-cell interactions term, remains challenging, however. This could be due to our limited knowledge of such multi-cellular processes in comparison to intracellular pathways. In addition, cell-cell interactions may be caused by a diversity of biological contexts and processes; for example, it is intrinsically challenging to differentiate simple cell-type co-occurrence from more specific molecular interactions. As emerging technologies provide even richer and large datasets, methods such as SVCA will allow for a more fine-grained interpretation of signatures of cell-cell interactions. More hypothesis-driven research, possibly with simpler biological systems with clear positive and negative controls, can be instrumental toward this goal.

Although we have tested the calibration and robustness of SVCA, the model is not free of limitations. At present, the model does not account for technology-specific noise and instead assumes Gaussian-distributed residuals, thus requiring suitable processing of the raw data such that these assumptions are sufficiently met (see the STAR Methods). Further development could consider a generalized random effects model, for example to couple the random effect component with a negative-binomial likelihood. A second limitation of SVCA is that the model is univariate, which means that individual genes or proteins are modeled independently from each other. Multivariate extensions could account for relationships between genes involved in the same pathways, either in an unsupervised manner or using prior knowledge (Buettner et al., 2017). Such approaches could provide a more comprehensive understanding of how biological processes are affected by tissue structure. Additionally, extensions could include modeling interactions between environmental and cell-cell interaction effects, which are treated as independent additive factors at present. As the size of the spatial expression dataset increases with the development of higher-throughput technologies, scalability will also become an important challenge for SVCA. The computational cost scales linear in the number of genes, and massive parallelization can be obtained with adequate computational infrastructure. Also, the random effect approach typically scales cubically in the number of cells, which can be circumvented by splitting bigger images into multiple patches and averaging the resulting SVCA signatures. In future work, faster inference schemes based on sparse approximations (Hensman et al., 2013, Quiñonero-Candela and Rasmussen, 2005, Snelson and Ghahramani, 2006) or random feature selection (Rahimi and Recht, 2008, Oliva et al., 2016). Future work will focus on developing these features.

There is a growing appreciation of the role of spatial distribution of proteins, transcripts, and other molecules in determining tissue functioning and its deregulation in disease, with potential value as predictors of clinical outcomes (Bodenmiller, 2016). This is largely driven by vigorous development of novel technologies that enable us to capture such data (Bodenmiller, 2016, Lin et al., 2017, Goltsev et al., 2018, Aichler and Walch, 2015, Schulz et al., 2018). Future datasets at increased scale and resolution will enable powerful applications of the SVCA framework, which we have presented in this manuscript.

## STAR★Methods

### Key Resources Table

**Table:** 

REAGENT or RESOURCE	SOURCE	IDENTIFIER
**Deposited Data**		

IMC data	Schapiro et al., 2017	https://www.nature.com/articles/nmeth.4391
seqFISH data	Shah et al., 2017	https://www.ncbi.nlm.nih.gov/pmc/articles/PMC5087994/

**Software and Algorithms**		

SVCA	this paper	https://github.com/damienArnol/svca
Limix	Lippert et al., 2014	https://github.com/damienArnol/svca

### Lead Contact and Materials Availability

Further information and requests for resources and reagents should be directed to and will be fulfilled by the Lead Contact, Oliver Stegle (Oliver.stegle@embl.de).

### Method Details

#### SVCA Model Overview

SVCA uses a random effect approach based on the Gaussian Process (GP) framework (Rasmussen and Williams, 2006, Lippert et al., 2014) using additive covariance functions. The covariance is composed of four terms, modeling our assumption that the variance across cells of the gene expression level is due to three additive effects: an intrinsic effect due to the cell state, a cell-cell interaction effect, due to the state of the neighboring cells, and an environmental effect due to unobserved factors in the cell micro environment, such as local access to oxygen, nutrients etc.

In the following, we will define the different terms of the covariance, show how they are parameterized and how these parameters are optimized. We will then explain how this model can be used to assess the proportion of variance explained by each effect, as well as the statistical significance of each effect. See also Figure S1.

We will rely on the following nomenclature and notations:

Nomenclature:

• Molecule of interest: Individual molecule, typically a gene or protein, on which SVCA is fitted.
• Cell state: Intrinsic characteristic of a cell. In this paper, we take the overall expression profile excluding the gene or protein of interest as a multidimensional and continuous measure of cell state. Other possibilities include classifying cells into cell types.
• Cellular neighborhood composition: Continuous measure of the molecular composition of a cell’s neighboring cells, summarized by weighting the molecular profiles of all neighboring cells with a squared exponential function of their distance to the focal cell.
• Intrinsic effect: Effect of the cell state on the expression level of the molecule of interest.
• Cell-cell interaction effect: Effect of cell-cell interactions on the expression level of the molecule of interest. These interactions may account for signaling between cells but also cell-types cooccurrences for example.
• Environmental effect: Effect of the cell’s position on the expression level of the molecule of interest. This effect accounts for unmeasured variables from the microenvironment with an effect on gene expressions, such as local glucose or oxygen access.
• Spatial variance signature: Concatenation of all variance estimates (intrinsic effect, environmental effect, cell-cell interaction effect and residual noise) across all molecules for a given image.

Notations:

• N - number of cells in a given image
• D - number of molecules (eg genes or proteins) in a given image
• Y - Expression level of the molecule of interest in all cells Dimensions: N×1
• X - Cell state matrix made of the entire expression profile of each cell minus the molecule of interest. Dimensions: N×(D−1). The molecule of interest is removed from the cell state matrix to prevent any cell-cell interaction false positive due to signal spillover between cells, as well as trivial intrinsic effect.
• $d_{i,j}$ - euclidean distance between cell i and j
• $K_{int}$ - cell-cell covariance for the intrinsic effect. Dimensions: N×N
• $K_{c-c}$ - cell-cell covariance for the cell-cell interaction effect. Dimensions: N×N
• $K_{env}$ - cell-cell covariance for the environmental effect. Dimensions: N×N

With these notations, SVCA models the expression level Y across cells with the following Gaussian Process model:

$$Y=N\left(0,K_{int\;}+K_{c-c}+\;K_{env}+\;\sigma_{\epsilon }^{2}I_{n\;}\right)$$

#### Definition of Covariance Terms

The intrinsic effect is the effect of the cell state on the expression level of the molecule of interest. In our framework, it is modeled with the linear covariance term $K_{int\;}=\sigma_{I}^{2}XX^{T}$. This covariance term corresponds to a Bayesian linear regression that models the effect of the cell expression profile on the expression of the gene of interest: $Y_{i\;}=\sum_{d\;\neq \;c}X_{i,d}\beta_{d}^{int}$, where c denotes the index of the gene of interest, with the following Normal prior on the effect sizes: $\beta^{int}∼N\left(0,\sigma_{int}^{2}\;I\right)$. This covariance function has a scaling hyperparameter $\sigma_{int}^{2}$, which is proportional to the int variance explained by this component.

The environmental effect aims at accounting for other local sources of variation in the cell micro-environment which are not measured in the data and have an effect of the expression level of the modeled gene. To model this unobserved source of variation, we consider a Squared Exponential Kernel $K_{env}=\sigma_{E}^{2}\;exp\left(-d_{i,j}^{2}/2l^{2}\right)$, which is able to capture complex non-linear dependencies and has previously been applied to spatial expression data (Svensson et al., 2018).

The cell-cell interaction effect models the effects of the types or states of all neighboring cells on the expression level of the molecule of interest. In the GP framework, it is modeled with the covariance function: $K_{c-c\;}=\sigma_{c-c}^{2}ZXX^{T}Z^{T}$where $Z_{i,j}=f\left(d_{i,j}\right)=exp\left(-d_{i,j}^{2}/2l^{2}\right)$for every couple of cells i and j. $Z_{i,i}=0$for all i. This covariance term is equivalent to a Bayesian linear regression where gene expression profiles of all neighboring cells are used as covariates and the effect of a cell i on a cell j is weighted by a function of the distance between them: $Z_{i,j}$.

#### Parameters Inference

The variance parameters of the SVCA model are optimized by maximizing the log likelihood of the data using a gradient-based optimizer (Rasmussen and Williams, 2006, Lippert et al., 2014):

$$L=logP\left(y|\sigma_{I},\sigma_{c-c},\sigma_{E},l\right)$$

$$=-1/2\;Y^{T\;}\cdot K\left(\sigma_{I},\sigma_{c-c},\sigma_{E},l\right)\cdot Y-1/2\;log|K\left(\sigma_{I},\sigma_{c-c},\sigma_{E},l\right)|-N/2\;log2\pi$$

Where $K\left(\sigma_{I},\sigma_{c-c},\sigma_{E},l\right)=K_{int}\left(\sigma_{I}\right)+K_{c-c}\left(\sigma_{c-c},l\right)+K_{env}\left(\sigma_{E},l\right)+\sigma_{\epsilon }^{2}\;I_{n}$

The scales of the covariance terms, $\sigma_{I},\sigma_{c-c},\sigma_{E}$ and $\sigma_{\epsilon }$ are optimized with gradient descent using the lbfgs optimizer, which updates the parameters iteratively through small steps along the gradient of the likelihood until it reaches a local optimum (null gradient). The length scale of the environmental and the local terms is optimized with a grid search strategy, which avoids possible local optima.

#### Estimates of Variance Components

Variance components for each effect are estimated using Gower factors $G\left(K_{effect}\right)$ (Searle, 1982, Kostem and Eskin, 2013):

$$G\left(K_{effect}\right)=\frac{tr\left(PK_{effect}P\right)}{n-1}\;\mathrm{with}\;P=I_{n}-J_{n}$$

The Gower Factor of a covariance term computes the expected variance of a random variable which is normally distributed with the considered covariance. In other words, the Gower factor of each covariance term of the SVCA model computes the amount of gene/protein variance across cells which is explained by the corresponding effect:

For Y∼N(0,K), G(K)=E[Var(Y)]

To compute the fraction of variance explained by each effect modeled in SVCA (intrinsic, environmental, cell-cell interactions and noise), we normalize Gower factors as follows

$$Var_{eff\;}=\frac{G\left(K_{eff}\right)}{\sum_{other\;effects}G\left(K_{eff}\right)+G\left(\sigma_{\epsilon }^{2}I_{n}\right)}$$

This procedure enables us to break down the variance of every protein, across cells in the three effects of interest plus the noise.

#### Comparison to Related Models

##### Schapiro et al. (2017) - HistoCAT

HistoCAT(Schapiro et al., 2017) aims at measuring spatial co-occurrence of different cell types. Briefly, cells of one or multiple images are classified into discrete cell-types based on their expression profile using a clustering algorithm. For every cell, a neighborhood is defined as containing all cells within a fixed distance threshold (measured from membrane to membrane). Using this fixed neighborhood definition, histoCAT counts the number of occurrences of a given pair of cell types, in the same neighborhood. This number is then compared to a null distribution obtained from permuting the cells’ positions, which gives a p value for positive and negative cell types interactions.

Unlike SVCA, histoCAT does not quantify the effect of these interactions on individual expression levels.

##### Battich et al. (2015)

Battich et al. (2015) uses a regression approach to measure the effect of the cell microenvironment on individual expression levels. Briefly, 183 features are collected, quantifying intrinsic cell properties and microenvironmental properties. Microenvironmental features namely account for local cell crowding, number of adjacent neighbors, intercellular space around the cell, as well as the molecular profile of the neighbors, based on a fixed distance threshold. The dimensionality of this feature set is then reduced using principal component analysis (PCA), and single cell expression profiles are modeled with a fixed effect linear model with the first 20 PCs as covariates. The PCs are then *a posteriori* linked to the microenvironmental features of interest. Biological replicates are used to quantify the amount of variance explained by each covariate using out of sample prediction.

This method therefore quantifies directly the effect of microenvironmental features including cell-cell interactions. Unlike SVCA however, it relies on a definition of discrete microenvironmental features and the definition of fixed parameters such as a distance threshold to define a cell’s neighborhood, which limits the applicability of the method to general spatial data.

##### Goltsev et al. (2018)

Goltsev et al. (2018) approach also relies on the definition of discrete microenvironmental variables, used in a fixed effect linear model to predict the expression level of individual markers out of sample. In contrast to Battich et al., microenvironmental variables are not defined directly based on the molecular profile of neighboring cells, but based on the cell-type composition of the neighborhood. The different neighborhood cell-type compositions are clustered into discrete i-niches, used as a discrete input for the linear model.

This method therefore enables to quantify directly the effect of cell-cell interactions on individual molecular profiles of single cells. However, it again relies on *a priori* definition of microenvironmental variables, this time based on discrete cell-type assignments.

#### Model Validation Using Simulated Data

In order to be as realistic as possible, our simulations were based on real data from 11 images of the IMC dataset (Giesen et al., 2014): real cell positions, cell states, and intrinsic and environmental effects were used, and only the cell-cell interaction effect was rescaled for the purpose of the simulations.

Our workflow was as followed:

• Fitting the SVCA model to the real dataset considered here (11 images and 26 proteins)
• Simulating data from a multivariate normal distribution, with a covariance made of:
• the intrinsic covariance from the fitted model
• the environmental covariance from the fitted model
• the noise covariance from the fitted model
• a cell-cell interaction covariance which is a rescaled version of the one fitted to the data: $Y=N(0,{\hat{K}}_{int\;}+k_{sim}\times {\hat{K}}_{c-c}+{\hat{K}}_{env}+{\hat{\sigma }}_{\epsilon }^{2}I_{n}$, where $\hat{K}$ represents the fitted covariance terms
• Refitting SVCA to the simulated data, where the variance explained by cell-cell interactions is known from the rescaling step.
• Comparing the variance estimates for cell-cell interactions with the ground truth.

In the following, the proportion of variance attributable to cell-cell interactions in the simulated data ranged from 10% to 90% (x∈[0.1;0.9]), and the rescaling factor $k_{sim}$was chosen accordingly: $k_{sim}=\frac{x}{1-x}\times \frac{G\left({\hat{K}}_{int\;}+{\hat{K}}_{c-c}+{\hat{K}}_{env}+{\hat{\sigma }}_{\epsilon }^{2}I_{n}\right)}{G\left({\hat{K}}_{c-c}\right)}$

#### Out of Sample Prediction on Real Data

The prediction performance of alternative models was assessed using 5-fold cross-validation. In order to assess the utility of different covariance terms in the model, we considered the following models:

• a model with only an intrinsic covariance to it.
• a model with an intrinsic component and a local component.
• the full model with all three terms

Models were assessed by the mean prediction of Gaussian process regression (Rasmussen and Williams, 2006):

$$Y_{pred}=\hat{K}\left(X_{pred},X\right)\cdot {\left[\hat{K}+{\hat{\sigma }}_{\epsilon }^{2}I_{n}\right]}^{-1}\cdot Y$$

Where $\hat{K}={\hat{K}}_{int}+{\hat{K}}_{env}+{\hat{K}}_{c-c}$corresponds to the fitted covariance terms and ${\hat{\sigma }}_{\epsilon }$corresponds to the fitted noise scale. $\hat{K}\left(X_{pred},X\right)$corresponds to the fitted covariance function evaluated between the input for the hold-out sample $X_{pred}$ and the input for the training samples X.

#### Identifiability of Cell-Cell Interactions versus Environmental Effects

To understand the identifiability of cell-cell interactions versus environmental effects, we compared the variance estimates of SVCA with the variance estimates of a reduced model which does not account for cell-cell interactions. Both models were fitted in the simulation setting described in the main text (26 proteins, 11 images and 10 repeat experiments). Variance estimates of SVCA and the reduced model were averaged across proteins, images, and experiments.

Results were visualized using a Sankey plot (Figure 2E), which illustrates which term of the reduced model captures the variance that is explained by the cell-cell interaction term of the full model. The width of the edges correspond to an increase in the variance estimates for the intrinsic effect, the environmental effect and the noise, from the SVCA model to the reduced model. This represents the redistribution of the cell-cell interaction component to other variance estimate from the SVCA model to the reduced model.

#### Comparison to Baseline Models

In order to compare SVCA to simpler baseline approaches, we considered simulated data derived from a linear model which included an intrinsic effect, a cell-cell interaction effect and a confounding effect due to cell mis-segmentation.

As before, *in silico* gene expression profiles are generated from real IMC data. For a given IMC dataset, let X be the expression profiles across cells for all genes, of dimensions N×D. Let $d_{i,j}$ be the distance between cells i and j. We first simulated the expression profile of an *in silico* gene Y using the following linear model:

$$Y=\sqrt{\eta_{c-c}\;}Z\cdot X\beta_{c-c}+\sqrt{1-\eta_{c-c}}\left(X\cdot \beta_{I}+\epsilon \right)$$

Where $Z_{i,j}=f\left(d_{i,j}\right)=1/d_{i,j}^{2}$if the cell i is in the $N_{nn}$first neighbors of the cell j and $Z_{i,j}=0$otherwise.

The number of nearest neighbors involved in cell-cell interactions $N_{nn}$ was also varied in order to simulate cell-cell interactions of variable ranges. The effect sizes $\beta_{I}$ and $\beta_{c-c}$were drown from standard normal distributions, and the features X and Z⋅X were standardized such that the variance explained by cell-cell interactions is $\eta_{c-c}\in \left[0,1\right]$. ϵ is a standard Gaussian noise.

We then simulated mis-segmentation between neighboring cells. For every cell in the image, two cells were chosen as mis-segmented with the focal cell. The probability for a cell j to be mis-segmented with a cell i was taken from the probability vector:

$$p_{j}=\frac{1/d_{i,j}^{2}}{\sum_{j}1/d_{i,j}^{2}}\forall j$$

which models our assumption that the closer the cell, the more likely it is that they are mis-segmented.

The expression profile Y was then perturbed by mis-segmentation in the following manner:

$${\tilde{Y}}_{i}=\sqrt{1-\eta_{mis}\;}Y_{i\;}+\sqrt{\eta_{mis}\;\;}mean_{j}\left(Y_{j}\right)$$

where $mean_{j}\left(Y_{j\;}\right)$ is the mean of the expression profile Y in the cells which are mis-segmented with the cell i. Simulations are then done while varying the relative effect of mis-segmentation $\eta_{mis}$.

The X matrix was perturbed accordingly:

$${\tilde{X}}_{i,:}=\sqrt{1-\eta_{mis}}X_{i,:\;}+\sqrt{\eta_{mis}}mean\left(X_{j,:}\right)$$

This models our assumption that all genes are affected in the same way by mis-segmentation which is reasonable but does not account for different subcellular localization of genes.

We then compared SVCA to four simpler models using data generated from the simulation setting described above. All models accounted for a cell intrinsic effect on the expression level of the simulated gene and a cell-cell interaction effect. The first three models were linear regressions with Ridge regularisation. The coefficient of regularisation was learnt with cross-validation using the RidgeCV function from the scikit-learn package (Pedregosa et al., 2011) with default parameters.

In all three linear regression models, the intrinsic effect was modeled as a linear combination of the expression profile of all genes measured in the cell, excluding the gene of interest. The three models differed in how they accounted for cell-cell interactions. The first model used all cells in the image, the impact of each cell being weighted by a function of the distance to the focal cell $f\left(d_{i,j}\right)=1/d_{i,j}^{2}$. The second model considered the average expression profile of the 5 nearest neighboring cells. And the third linear regression took a weighted average of these five nearest neighbors with the same weighting function $f\left(d_{i,j}\right)=1/d_{i,j}^{2}$.

The fourth model which was compared to SVCA was a reduced GP model containing all the covariance terms of SVCA apart from the local effect.

#### Data Processing and Experimental Procedures

##### Imaging Mass Cytometry (IMC) Data

With IMC, the analyzed tissue or cell culture is laser-dissected into a subcellular resolution grid of so-called voxels of dimension 1 μm × 1 μm. Every voxel of this grid is then analyzed with cyTOF (antibody based method), which results in protein counts of 26 proteins per voxel, which can be aggregated into single cell counts after cell segmentation (Giesen et al., 2014, Sommer et al., 2011, Schüffler et al., 2015, Carpenter et al., 2006). We analyzed a dataset of 46 breast cancer biopsies imaged with Imaging Mass Cytometry coming from 23 patients (Schapiro et al., 2017) (6 images were removed from the original dataset as they exhibited one or multiple markers with zero variance). 38 of these images are associated to clinical data:

•ERstatus•PRstatus•Her2status•Grade•Biopsylocation(peripheryorcentre)

These images contain between 267 and 1455 cells, with an average around 900 cells. 26 proteins counts are quantified at a subcellular level (between 10 and 100 pixels/measurements per cell).

The single cell expression levels were computed by taking the median protein count across pixels.

##### Data processing

In all cases, the data were then transformed with an Anscombe’s trans formation for variance stabilization of Negative Binomial data (Anscombe, 1948). The dispersion parameterϕ in $\sigma^{2\;}=\mu +\phi \times \mu^{2}$ is optimized with gradient descent and the following log transformation is applied to the data:

y=log(x+1/2ϕ)

The resulting signal is then normalized by regressing out the log of the total signal in the cell. This last step aims at taking into account local batch effects which would make some cells ”brighter” overall.

Before fitting SVCA, the stabilized expression profile of the target gene Y is subsequently raked standardized and transformed into normally distributed data using the probit function, in order to ensure a more robust fitting process due to a lesser sensitivity to outliers.

##### Analysis

We fitted SVCA on all processed IMC images independently and validated the results using 5-fold cross-validation as described in the *Model Validation* section. We then performed Principal Component Analysis on all SVCA variance signatures and used the *ClusterSignificance* R package to quantify tumor grade separation in the Principal Component space, as explained in the *Downstream Analysis* section.

##### mer-FISH and seq-FISH Data

Although mer-FISH and seq-FISH techniques differ slightly, the data produced and available online (Moffitt et al., 2016, Shah et al., 2017) come in a similar format. Briefly, it comes as a list of detected individual RNA molecules, associated to a precise position on the tissue and the index of the cell each molecule belongs to (obtained with automatic cell segmentation). Summarizing this data into a molecule count at the single cell level is therefore straightforward.

We analyzed a mer-FISH dataset of 20 images taken on a single plate of breast cancer cell culture. Each image contained between 2500 and 2900 cells and 130 genes were measured. Additionally, we analyzed a seqFISH dataset consisting of 20 images of a single mouse hippocampus (Shah et al., 2017). The images were taken in different regions of the hippocampus and 249 genes were measured.

##### Analysis

We fitted SVCA on all processed seq-FISH images independently and validated the results using 5-fold cross-validation as described in the *Model Validation* section. We then performed Principal Component Analysis on all SVCA variance signatures and Gene Set Enrichment Analysis for the genes with higher cell-cell interaction components, as described in the *Downstream Analysis* section.

#### Downstream Analysis

##### Gene Categories Enrichment in seqFISH

The statistical significance for the enrichment of gene categories for cell-cell interactions and intrinsic effect was done using a permutation strategy similar to the one used in GSEA (Subramanian et al., 2005, Mootha et al., 2003):

• Genes were ranked based on the size of the tested variance component (cell-cell interactions or intrinsic effect)
• A GSEA-like trace was computed for each gene category and the height of this trace is considered as a test statistic.
• Gene names were permuted 10,000 times in order to estimate an empirical p value for the statistic described above.
• p values were adjusted for multiple testing using a Benjamini-Hochberg procedure (Benjamini and Hochberg, 1995).

##### Grade Separation in Principal Component Analysis

We used the Mlp method of the ClusterSignificance package in R (Serviss et al., 2017) to quantify the separation between grades 1 and 3 in the PCA projection of the SVCA signatures.

Briefly, the package computes the centroids of the principal components for the tumors of grade 1 and 3 independently, and projects the samples onto the line going between the two centroids, providing a one dimensional representation of the samples. The package then computes the following separation score between the grade 1 and grade 3: $score=1-\sqrt{{\left(1-specificity\right)}^{2\;}+{\left(1-sensitivity\right)}^{2}}$, using the perpendicular line that best separates the two classes in their one dimensional representation.

Finally, ClusterSignificance uses permutations to compute the null distribution of the score defined above and deduce a quantile-based p value for the separation between grade 1 tumors and grade 3 tumors.

### Quantification and Statistical Analysis

#### Significance of Variance Components

This section describes the procedure to assess the significance of the cell-cell interaction component, as this is the variance component of main interest for our study. The significance of other variance components in SVCA can be assessed analogously.

The significance of the cell-cell interaction component is assessed based on the log likelihood ratio (LLR) between the full SVCA model and a reduced model omitting the cell-cell interaction component. Given that the reduced model is nested, we rely on Wilks’ theorem (Wilks, 1938), where if the null hypothesis is true (no cell-cell interactions), the LLR statistics is expected to follow a $\chi^{2}$ distribution. In practise, we calibrate this $\chi^{2}$ distribution by fitting its parameter to an empirical null distribution of LLRs obtained from simulations (Bůžková et al., 2011, Casale et al., 2017).

The simulation procedure is as follows. For all proteins and all images, we fitted the null model (no cell-cell interaction) and simulated data from the fitted normal distribution. We simulated 100 data points for each test and then fitted a $\chi^{2}$ distribution to those using an off-the-shelf non linear optimization method (“[PDF]Package ‘Nloptr’ - CRAN.R-Project.org,” n.d.). We then compared the LLR obtained, for each protein and each image, for the real data to the corresponding fitted $\chi^{2}$ distribution and estimated p values from this comparison.

For every test, we computed a cell-cell interaction p value using the method described before and used the Benjamini-Hochberg procedure (Benjamini and Hochberg, 1995) to adjust p values for multiple testing. For each protein, we then counted the number of images in which the cell-cell interaction component was significant for a FDR threshold of 1%.

#### P Value Calibration for the Cell-Cell Interaction Component

We used simulations to assess the p value calibration for the cell-cell interaction component. For 11 random images and all 26 proteins of the IMC dataset, we simulated from the null model (SVCA without the cell-cell interaction term whose parameters were fitted to the data). We then used the procedure described above to compute p values for the null distribution, and computed the empirical false positive rate for multiple p value thresholds (Figure 2).

#### Error Bars and Boxplots

Error bars correspond to plus and minus one standard deviation across images and proteins (Figure 3). The lower and upper hinges of boxplots correspond to the 25th and 75th percentiles. The lower and upper whiskers extend from the hinge to the largest value no further than 1.5 ^∗^ IQR (Inter Quantile Range) from the lower and upper hinge respectively.

### Data and Software Availability

An open source implementation of SVCA is available at https://github.com/damienArnol/svca, which builds on the limix package (Lippert et al., 2014).

## Supplemental Information

Document S1. Figures S1–S4

Table S1. Manual Gene Classification for the Hippocampus RNA Dataset (seqFISH Data), Related to Figure 4 The first column corresponds to the gene name as given in Shah et al. (2017). The second column corresponds to the manual classification of the gene.

Document S2. Article plus Supplemental Information
