# Supplementary material for: Modeling Cell-Cell Interactions from Spatial Molecular Data with Spatial Variance Component Analysis
Source: Cell Rep. 2019 Oct 1;29(1):202–211.e6. doi: 10.1016/j.celrep.2019.08.077 (PMC6899515; doi:10.1016/j.celrep.2019.08.077)
Supplement: Document S1. Figures S1–S4 [file mmc1.pdf]

Cell Reports, Volume *29*

Supplemental Information

Modeling Cell-Cell Interactions
from Spatial Molecular Data
with Spatial Variance Component Analysis

Damien Arnol, Denis Schapiro, Bernd Bodenmiller, Julio Saez-Rodriguez, and Oliver Stegle

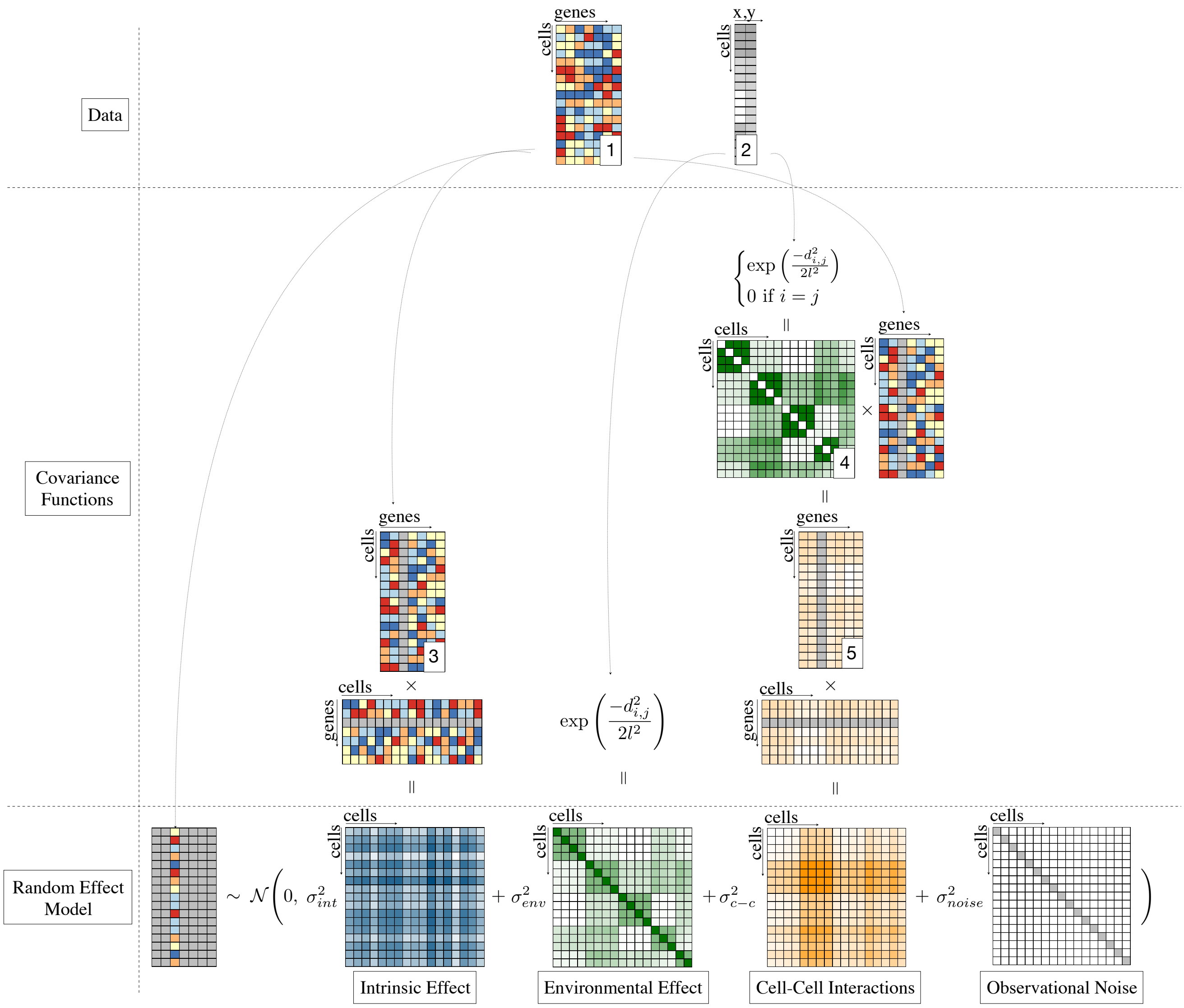

Figure S1 | SVCA model definition. Related to Figure 1.

SVCA takes as an input single cell expression data as a cell times gene/protein matrix (1) and the spatial location of the cells as a cell times (x,y) coordinates matrix (2). Individual genes are modelled as multivariate normally distributed, with additive covariance components that account for different effects modelled by SVCA (intrinsic, environmental and cell-cell interactions, **Fig.1**). The covariance for intrinsic effects is computed as the empirical covariance of the expression profiles between cells, where the modelled gene has been removed from the expression matrix (3). Environmental effects are modelled using a Squared Exponential covariance defined on the relative distance between cells. Cell-cell interactions are modelled using a cellular neighbourhood matrix (5) which aggregates, for each cell, the molecular composition of the neighbouring cells. This is achieved by weighting the molecular profiles of all neighbouring cells with a squared exponential function of the distance. This can be written as a product between a squared exponential covariance matrix whose diagonal elements were removed (4) and the expression matrix (3). The final cell-cell interaction covariance is computed as the empirical covariance of the cellular environment matrix between cells. SVCA's training then consists in learning the scale of every covariance term.

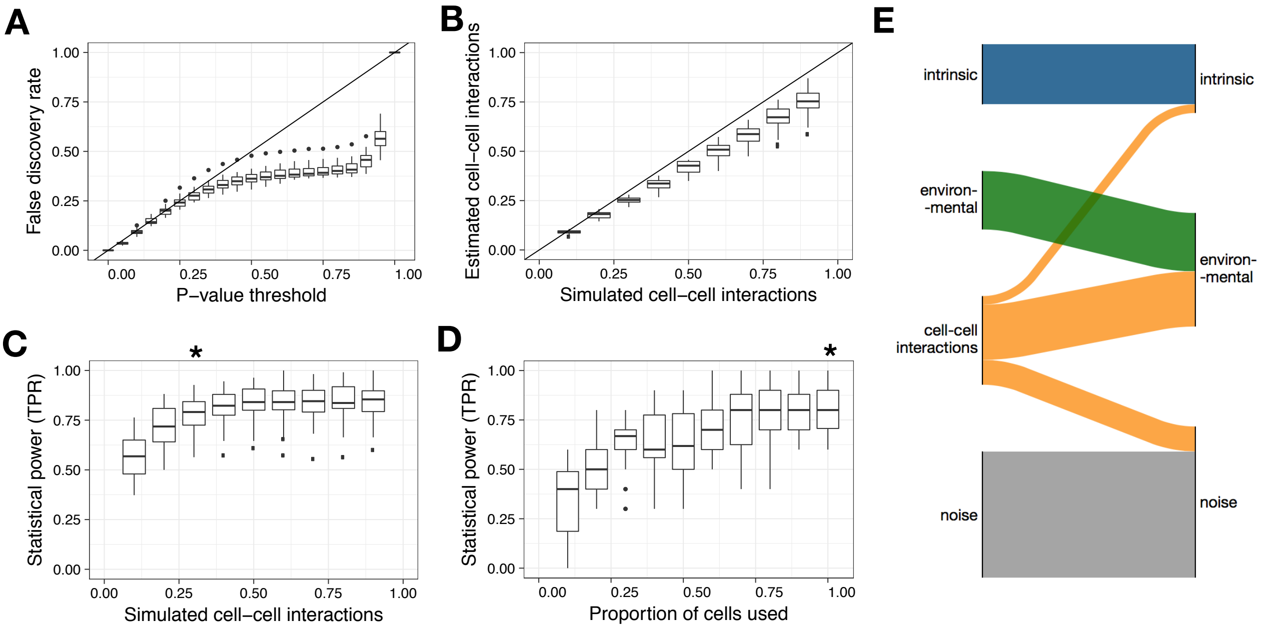

Figure S2 | Simulations from the generative model demonstrates SVCA's conservativity and statistical power. Related to Figure 2.

(a) Empirical false discovery rate for the cell-cell interaction test using data simulated from the null, without cell-cell interactions. Shown is the empirical false discovery rate (FDR) as a function of the P value threshold. **(b)** Fraction of variance due to cell-cell interactions estimated by SVCA when varying the true fraction of variance explained by cell-cell interactions (**Methods**). **(c,d)** Statistical power of the test for cell-cell interactions (at family-wise error rate <1%), when varying the simulated fraction of variance explained by cell-cell interactions (**c**) and when considering different subsets of cells from the full dataset for model fitting (**d**). The full dataset with all cells was used for model fitting in **c** (indicated using the asterix symbol). In each panel, boxplots display the distribution of results across 26 proteins. Rates in panel **a**, **c**, **d** (True Positive Rate - TPR and False Discovery Rate - FDR) are computed for each protein, aggregated across 110 simulations (11 images times 10 repeat experiments). Similarly, panel **b** depicts average variance estimates across the same set of 110 simulations for each protein. (**e**) Sankey plot displaying how the variance explained by cell-cell interactions is captured by the other terms when omitting interaction effects in the model. Bar height denotes estimated variance fractions (lef: SVCA; right: reduced model). Linking edges indicate the redistribution of variance estimates between the full and the reduced model. Both models were fitted on the same simulated data for 11 images and 26 proteins with cell-cell interactions explaining 30% of the variance of simulated expression levels and results shown are averaged across images and proteins. (**Methods**).

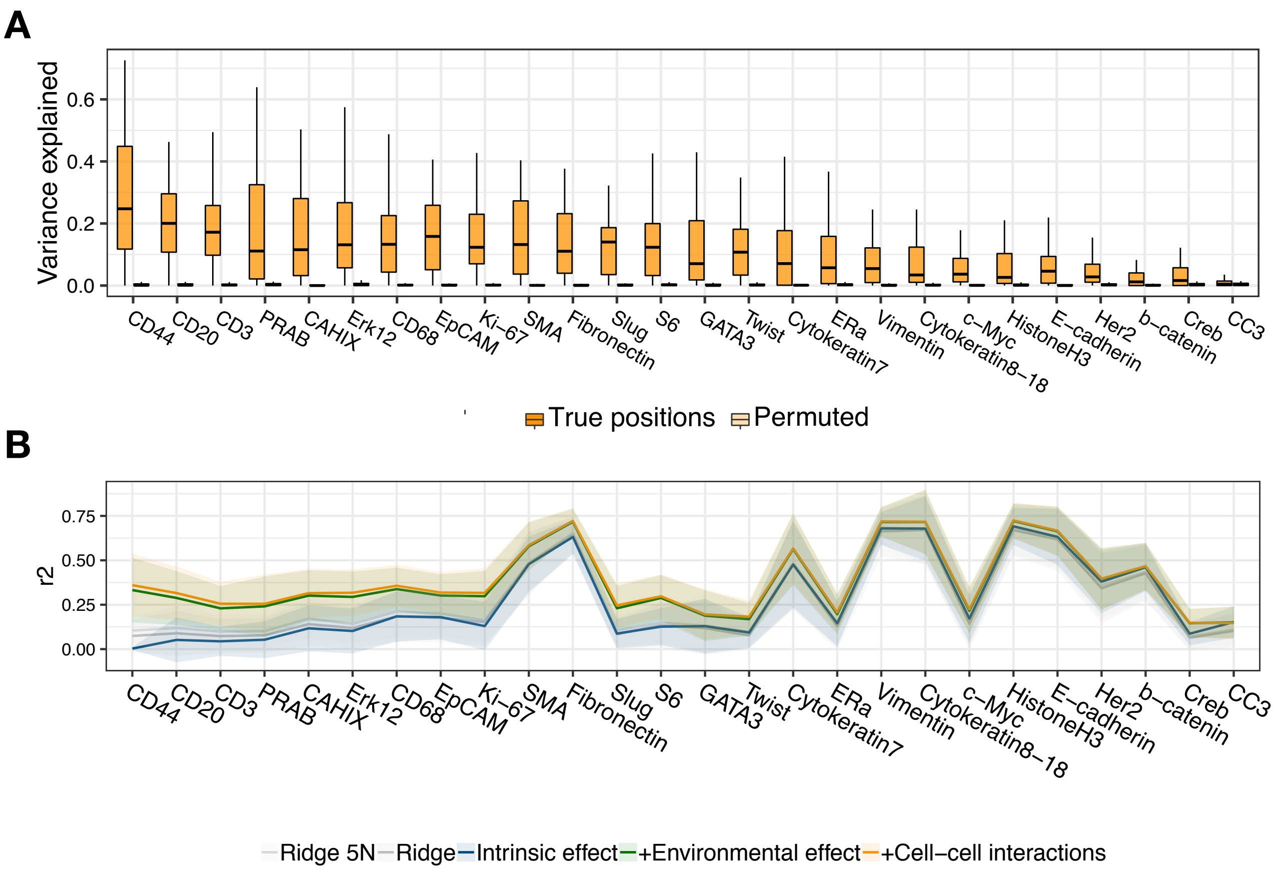

Figure S3 | IMC validation. Related to Figure 3.

(a) Cell-cell interaction variance components with true cell positions and permuted positions. Each boxplot contains one data point per IMC image. (b) Prediction accuracy for SVCA and alternative models using 5-fold cross-validation. The blue and green lines correspond to two reduced Gaussian Processes including respectively an intrinsic component only, and both an intrinsic and a local component. The two grey lines correspond to alternative linear regressions (**Methods**). Results are shown for the 20 genes with highest cell-cell interactions. The solid lines correspond to the coefficients of determination between predicted gene expression and observed values. The shaded areas correspond to plus and minus one standard deviation across images.

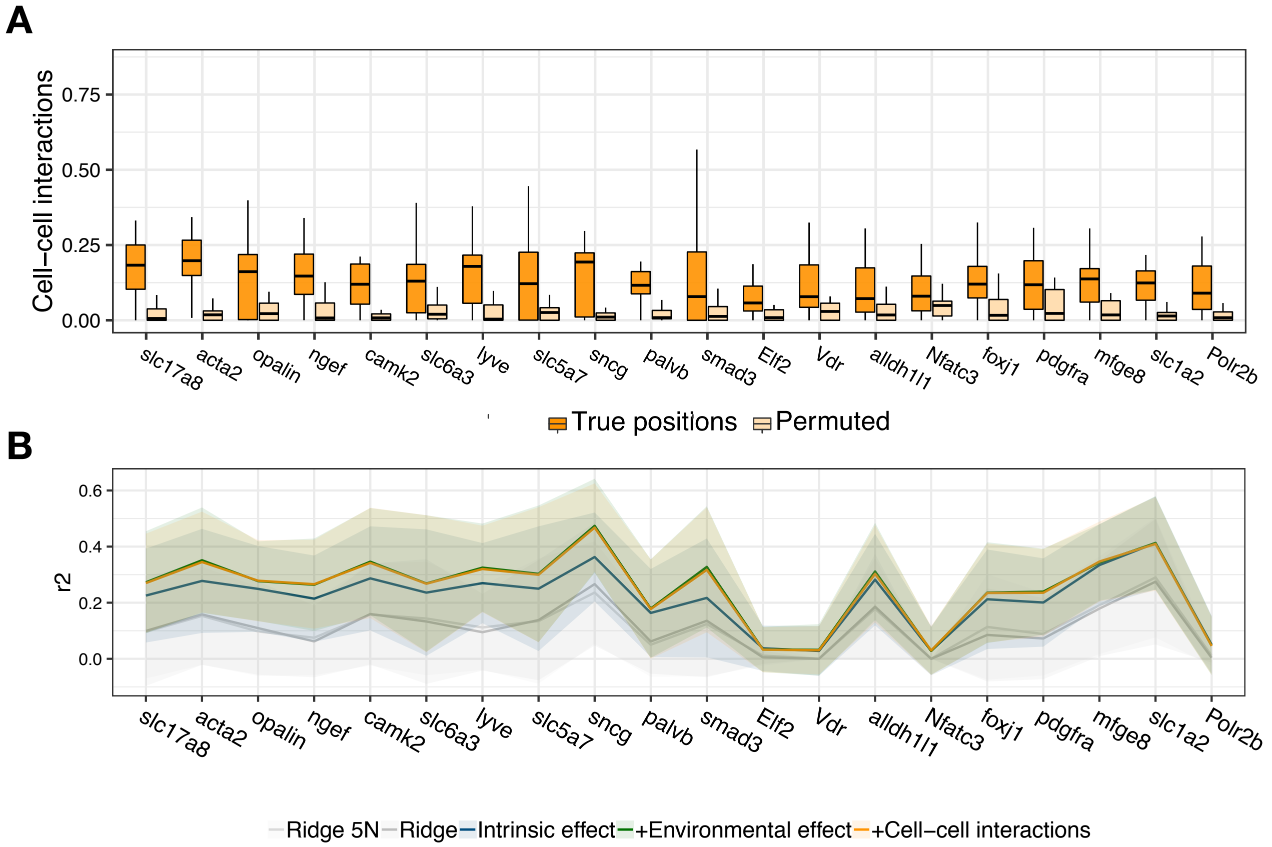

Figure S4 | seqFISH validation. Related to Figure 4.

(a) Cell-cell interaction variance components with true cell positions and permuted positions. Each boxplot contains one data point per seqFISH image. (b) Prediction accuracy for SVCA and alternative models using 5-fold cross-validation. The blue and green lines correspond to two reduced Gaussian Processes including respectively an intrinsic component only, and both an intrinsic and a local component. The two grey lines correspond to alternative linear regressions (**Methods**). Results are shown for the 20 genes with highest cell-cell interactions. The solid lines correspond to the coefficients of determination between predicted gene expression and observed values. The shaded areas correspond to plus and minus one standard deviation across images.
